# Synthesis, structural characterization and study of antioxidant and anti-PrP^Sc^ properties of flavonoids and their rhenium(I)–tricarbonyl complexes

**DOI:** 10.1007/s00775-022-01986-9

**Published:** 2023-01-25

**Authors:** Pigi Glykofridi, Vassiliki-Eleni Tziouri, Konstantinos Xanthopoulos, Maria-Eirini Vlachou, Susana Correia, Anna-Lisa Fischer, Katrin Thüne, Antonios Hatzidimitriou, Inga Zerr, Matthias Schmitz, Theodoros Sklaviadis, Dimitra Hadjipavlou-Litina, Dionysia Papagiannopoulou

**Affiliations:** 1 Laboratories of Pharmaceutical Chemistry and of Pharmacology, School of Pharmacy, Faculty of Health Sciences, Aristotle University of Thessaloniki, 54124 Thessaloniki, Greece; 2 Department of Neurology, University Medical Center Göttingen and the German Center for Neurodegenerative Diseases (DZNE), Göttingen, Germany; 3 Laboratory of Inorganic Chemistry, Department of Chemistry, Faculty of Sciences, Aristotle University of Thessaloniki, 54124 Thessaloniki, Greece

**Keywords:** Rhenium complexes, Flavonoids, Anti-oxidant, Lipid peroxidation, Lipoxygenase, Prion diseases

## Abstract

This study aims at the synthesis and initial biological evaluation of novel rhenium–tricarbonyl complexes of 3,3′,4′,5,7-pentahydroxyflavone (quercetin), 3,7,4΄-trihydroxyflavone (resokaempferol), 5,7-dihydroxyflavone (chrysin) and 4΄,5,7-trihydroxyflavonone (naringenin) as neuroprotective and anti-PrP agents. Resokaempferol was synthesized from 2,2΄,4-trihydroxychalcone by H_2_O_2_/NaOH. The rhenium–tricarbonyl complexes of the type *fac*-[Re(CO)_3_(Fl)(sol)] were synthesized by reacting the precursor *fac*-[Re(CO)_3_(sol)_3_]^+^ with an equimolar amount of the flavonoids (Fl) quercetin, resokaempferol, chrysin and naringenin and the solvent (sol) was methanol or water. The respective Re–flavonoid complexes were purified by semi-preparative HPLC and characterized by spectroscopic methods. Furthermore, the structure of Re–chrysin was elucidated by X-ray crystallography. Initial screening of the neuroprotective properties of these compounds included the in vitro assessment of the antioxidant properties by the DPPH assay as well as the anti-lipid peroxidation of linoleic acid in the presence of AAPH and their ability to inhibit soybean lipoxygenase. From the above studies, it was concluded that the complexes’ properties are mainly correlated with the structural characteristics and the presence of the flavonoids. The flavonoids and their respective Re-complexes were also tested in vitro for their ability to inhibit the formation and aggregation of the amyloid-like abnormal prion protein, PrP^Sc^, by employing the real-time quaking-induced conversion assay with recombinant PrP seeded with cerebrospinal fluid from patients with Creutzfeldt–Jakob disease. All the compounds blocked de novo abnormal PrP formation and aggregation.

**Graphical abstract:**

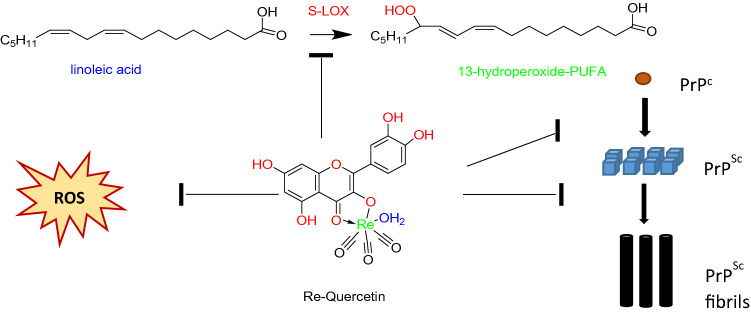

**Supplementary Information:**

The online version contains supplementary material available at 10.1007/s00775-022-01986-9.

## Introduction

Neurodegenerative disease is currently the seventh leading cause of mortality especially among older people worldwide, and Alzheimer’s disease (AD) contributes to approximately 60–70% of the cases. Neurodegenerative diseases are typically defined by specific protein accumulations and are classified, as amyloidoses, tauopathies, a-synucleinopathies, and transactivation response DNA binding protein 43 (TDP-43) proteinopathies. In addition, neurodegenerative diseases share other pathological features, such as progressive neuronal dysfunction and death, which are associated among others with oxidative stress and neuroinflammation [1, 2].

Amyloidoses are characterized by insoluble amyloid fibrous proteins that have specific structural characteristics, including a rich *β*-sheet secondary structure, which permits binding of fluorescent dyes, such as Thioflavin T and Congo red. In AD *β*-amyloid (Aβ) peptide deposits are formed as a proteolytic product of the amyloid precursor protein. In prion disease the pathogenic isoform of the prion protein (PrP^Sc^) is deposited in an amyloid-like formation, after conversion of the normal protein isoform (PrP_C_) to it abnormal counterpart. Studies have shown that once formed, the PrP^Sc^ behaves as a “seed” and the aberrant proteins propagate by acting as templates to corrupt other like molecules, produced physiologically in the cell. The prion concept has expanded to include many of the aberrant proteins that characterize neurodegenerative diseases [3, 4]. A proposed mechanism, in amyloidogenic disease, entails a process where the native protein forms folded or unfolded oligomers that accelerate aggregation to fibrils. In this light, therapeutic strategies targeted to the aggregation process have attracted a lot of interest and several agents have shown promising results in clinical trials [5].

Oxidative stress is the accumulation of reactive oxygen species (ROS) in the cells, causing cellular dysfunction and lipid peroxidation is the oxidative damage of lipids as a result of the action of ROS and both are conditions that contribute to the pathological features of neurodegenerative disorders [6]. A number of neuroprotective agents have shown to inhibit lipid peroxidation, and among these flavonoids have shown promising results [7–12]. Flavonoids are natural polyphenolic compounds ubiquitous in vegetables, fruits, tea and other plants that exhibit a wide variety of biological activities, rendering thus the flavone scaffold attractive in drug design [13]. In addition, polyphenols and natural flavonoids, as well as their synthetic derivatives, demonstrate inhibition of cholinesterase, of *β*-secretase as well as inhibition of toxic *β*-amyloid formation and, therefore, have attracted increasing attention in the development of multi-targeted therapeutic agents against neurodegeneration [14–25]. In addition, flavonoid-metal complexes were shown to exhibit anti-oxidative properties [26, 27]. Derivatized flavonoids and chemically related structures (aurones) were radiolabeled with fluorine-18, a radionuclide used in positron emission tomography as well as with technetium-99 m, a γ-emitting radionuclide used in single photon emission computerized tomography [28–30] and were evaluated as radiodiagnostic agents due to their affinity to bind to *β*-amyloid plaques.

Metal complexes play an important role in medicinal chemistry, especially in theranostic approaches [31, 32]. Facial rhenium (I)–tricarbonyl complexes are attracting interest as building blocks for pharmaceutical applications, due to their demonstrated kinetic stability, structural and substitution flexibility, and anticancer properties. [33–35] Re(I)–tricarbonyl complexes with polyaromatic ligands exhibit fluorescent properties with potential as fluorescent probes for fluorescence microscopy imaging [36–38]. Furthermore, rhenium can also be used for the development of multi-targeted complexes by coordination of different pharmacophores around the metal core that is extremely useful in neurodegenerative multifactorial diseases with complex biochemistry. [39, 40] In addition, due to the chemical similarity between rhenium and technetium, the rhenium(I)–tricarbonyl complexes can be used as prototypes for the development of analogous [^99m^Tc][Tc(I)–tricarbonyl complexes as SPECT radiodiagnostic agents.

**Fig. 1:**
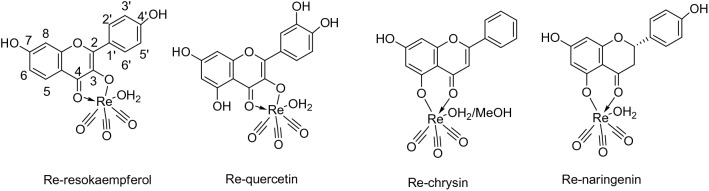
Structure of the Re–tricarbonyl–flavonoid complexes

In this work, the flavonoids 3,3′,4′,5,7-pentahydroxyflavone (quercetin), 3,7,4΄-trihydroxyflavone (resokaempferol), 5,7-dihydroxyflavone (chrysin) and 4΄,5,7-trihydroxyflavonone (naringenin) were complexed with rhenium–tricarbonyl (Fig. 1). The elucidation of the coordination site of quercetin with rhenium–tricarbonyl was supported by the characterization of Re–resokaempferol, Re–chrysin and Re–naringenin chelates. The complexes were evaluated for their anti-oxidant properties, for their ability to inhibit lipid peroxidation and to inhibit lipoxyhenase in comparison to the free flavonoids, since the role of oxidative stress and lipoxygenase’s pathway in neurodegenerative diseases is well known [41] Furthermore, the flavonoids and their rhenium complexes were evaluated for their ability to inhibit PrP^Sc^ formation and aggregation from human prion disease samples in cell-free assays.

## Experimental section

### General

All chemicals were reagent grade and the solvents for HPLC were HPLC grade. Re_2_(CO)_10_ was converted to Re(CO)_5_OTf as described in the literature [42, 43]. Soybean lipoxygenase, sodium linoleate, DPPH, and 2,2-azobis-(2-amidinopropane) dihydrochloride (AAPH) were obtained from Sigma Chemical, Co. (St. Louis, MO, USA).

IR spectra were recorded as KBr pellets on a Spectrum BX spectrophotometer (Perkin Elmer, Waltham, MA, USA) in the region 4000–500 cm^−1^. Elemental analysis was performed with ThermoFlash 2000 analyzer (Thermo Scientific, Waltham, MA, USA).^1^H and ^13^C NMR spectra were obtained on a DD2 Agilent 500 MHz spectrometer. ESI-MS data were obtained on an MSQ Plus™ LC/ MS (Thermo Scientific, Waltham, MA, USA). Hitachi F2000 Fluorospectrometer was used for emission spectra. HPLC analyses were performed on an Agilent HP 1100 series pump (HP, Waldbronn, Germany), connected to an HP 1100 multiple wavelength photodiode array detector. RP-HPLC analyses of the rhenium complexes were performed on an Agilent Eclipse XDB C18 column (25 cm × 4.6 mm, 5 μm) by applying a binary gradient method of Solvent A: H_2_O and Solvent B: methanol at a flow rate of 1 mL/min; System I: 100% of A initial composition that linearly converted to 25% A–75% B over 15 min, then to 5% A–95% B over 5 min. The composition was constant from 20 to 25 min at 95% B, before returning to initial conditions. Purification of the rhenium complexes was performed by semi-preparative HPLC on an Eclipse XDB C18 column (25 cm × 9.4 mm, 5 μm) by applying a binary gradient method of Solvent A: H_2_O and Solvent B: methanol at a flow rate of 2 mL/min; System II: 50% of A initial composition that linearly converted to 25% A–75% B over 15 min, then to 5% A–95% B over 5 min. The composition was constant from 20 to 25 min at 95% B, before returning to initial conditions. System III: 60% of A initial composition that linearly converted to 25% A–75% B over 15 min, then to 5% A–95% B over 5 min. The composition was constant from 20 to 25 min at 95% B, before returning to initial conditions. The antioxidant properties of the compounds were measured on a PerkinElmer lambda 20 (PerkinElmer Inc., Waltham, MA, USA) or on a Shimadzu, UV-1700 Spectrophotometer (Shimadzu Corp, Kyoto, Japan).

### Synthesis of 3,7, 4′-trihydroxyflavone (resokaempferol)

Adapted from the literature [44, 45], to a methanolic solution of 2,2',4-trihydroxychalcone (0.5 mmol), NaOH 2 M (3 mL) was added. A 30% aqueous solution of H_2_O_2_ (225 μL) was then added dropwise and the reaction mixture was stirred at room temperature for 24 h. The mixture was acidified by addition of HCl 5 N and the yellow solid received was filtered and recrystallized from methanol.

Yield: 24 mg (18%). IR (KBr, cm^−1^) 3309, 1611, 1560, 1508, 1277, 1244, 1181, 1120, 832.^1^H NMR (500 MHz, CD_3_OD) δ 8.11 (d, *J* = 8.4 Hz, 2H, H-2’/H-6’), 7.97 (d, *J* = 9.2 Hz, 1H, H-5), 6.91–6.88 (m, 4H, H-3’/H-5’, H-6, H-8). ^13^C NMR (126 MHz, CD_3_OD) δ 163.15, 163.15, 158.99, 157.13, 157.13, 146.08, 129.20, 126.03, 122.57, 114.87, 114.71, 113.89, 101.61. m.p. 305–308 ^0^C (lit. 310 ^0^C). ESI-MS (m/z) calc. for C_15_H_10_O_5_: M = 270.2; Found: [M-H]^−^ 269.01. Anal. calcd. for C_15_H_10_O_5_: C 66.67, H 3.73. Found: C 66.35, H 3.53.

### Synthesis of rhenium–tricarbonyl–flavonoid complexes

General method: A solution of Re(CO)_5_OTf (47 mg, 0.1 mmol) in H_2_O or MeOH (2 mL) was refluxed for 1 h to form the precursor [Re(CO)_3_(sol)_3_]^+^, (sol: H_2_O or MeOH) to which a methanolic solution (1 mL) of the flavonoid (0.1 mmol) and NaOH 1 M (0.1 mL) was added. The reaction mixture was refluxed for 4 h under nitrogen and stirred at room temperature for 18 h. The solvent was evaporated under reduced pressure and the product was purified by semi-preparative HPLC.

*Re–resokaempferol*: Yield 17 mg (60%). IR (KBr, cm^−1^) 3448, 2019, 1870, 1618, 1560, 1498,1458, 1420, 1277, 1175, 842. ^1^H NMR (500 MHz, CD_3_OD) δ 8.41 (d, *J* = 8.8 Hz, 2H, H-2’/H-6’), 8.05 (d, *J* = 8.9 Hz, 1H, H-5), 7.03 (s, *J* = 1.4 Hz, 1H, H-8), 7.00 (dd, *J* = 9.0, 1.7 Hz, 1H, H-6), 6.94 (d, *J* = 8.8 Hz, 2H, H-3’/H-5’). ^13^C NMR (126 MHz, CD_3_OD) δ 198.2, 181.6, 162.9, 159.1, 156.1, 150.91, 150.0, 129.2, 125.1, 123.3, 115.9, 115.0, 113., 101.01. ESI-MS (m/z): calc. for C_18_H_11_O_9_Re: *M* = 558.0; Found: [M–H_2_O–H]^−^ 538.77 (100%); 536.77 (60%). Anal. calcd. for C_18_H_11_O_9_Re·H_2_O: C 37.57, H 2.28. Found: C 38.08, H 1.99.

*Re–quercetin*: Yield 14 mg (46%). IR (KBr, cm^−1^) 3445, 2024, 1949, 1924, 1636, 1526, 1264, 1170, 700. ^1^H NMR (500 MHz, CD_3_OD) δ 8.07 (s, 1H, H’-2), 7.91 (d, *J* = 8.4 Hz, 1H, H’-6), 6.91 (d, *J* = 8.5 Hz, 1H, H’-5), 6.55 (s, 1H, H-8), 6.30 (s, 1H, H-6). ^13^C NMR (126 MHz, CD_3_OD) δ 197.7, 181.9, 164.3, 158.3, 155.8, 151.1, 148.7, 147.7, 144.7, 123.4, 120.3, 115.0, 114.4, 103.7, 98.8, 93.1. ESI-MS (m/z): calc. for C_18_H_11_O_11_Re: M = 590.0; Found: [M–H_2_O–H]^−^ 571.04 (100%); 569.05 (60%). Anal. calcd. for C_18_H_11_O_11_Re·H_2_O: C 34.56, H 2.42. Found: C 34.63, H 1.93.

*Re–chrysin*: Yield 15 mg (27%). IR (KBr, cm^−1^) 3386, 3200, 2021, 1876, 1631, 1518, 1424, 1168, 835, 768. ^1^H NMR (500 MHz, CD_3_OD) δ 7.97 (d, *J* = 7.5 Hz, 2H, H-2’/H-6’), 7.58–7.52 (m, 3H, H-3’/H-4’/H-5’), 6.87 (s, 1H, H-3), 6.32 (d, *J* = 1.9 Hz, 1H, H-8), 6.24 (d, *J* = 2.0 Hz, 1H, H-6). ^13^C NMR (126 MHz, CD_3_OD) δ 197.0, 196.9, 196.7, 178.1, 165.9, 165.4, 162.5, 159.2, 130.8, 128.9, 128.7, 126.0, 125.9, 108.2, 104.3, 104.2, 102.4, 102.2, 91.8, 91.6. ESI-MS (m/z): calc. for C_18_H_11_O_8_Re: M = 542.0; Found: [M–H_2_O–H]^−^ 522.78 (100%); 520.80 (60%). Anal. calcd. for C_18_H_11_O_8_Re·H_2_O: C 38.64, H 2.34. Found: C 38.50, H 2.11.

*Re–naringenin*: Yield 29 mg (51%). IR (KBr, cm^−1^) 3217(br), 2026, 1888, 1613, 1580, 1432, 1162, 1088, 832. ^1^H NMR (500 MHz, CD_3_OD) δ 7.30 (d, *J* = 8.0 Hz, 4H, H-2’/H-6’), 6.81–6.79 (m, 6H, H-3’/H-4’/H-5’), 5.87–5.86 (m, 2H, H-8), 5.73 (d, *J* = 1.9 Hz, 2H, H-6), 5.29 (d, *J* = 12.3 Hz, 1H, H-2**), 5.21 (dd, *J* = 12.6, 2.3 Hz, 1H, H-2*), 3.13 (dd, *J* = 16.9, 12.8 Hz, 1H, H-3*), 3.04 (dd, *J* = 16.9, 12.7 Hz, 1H, H-3**), 2.85–2.75 (m, 2H, H-3). ^13^C NMR (126 MHz, CD_3_OD) δ 197.4, 197.0, 196.7, 190.4, 168.5, 163.7, 163.7, 157.5, 129.7, 129.6, 127.6, 127.51, 114.9, 114.8, 99.2, 93.5, 78.0, 77.8, 42.1, 41.9. ESI-MS (m/z): calc. for C_18_H_13_O_9_Re: *M* = 560.0; Found: [M–H_2_O–H]^−^ 540.82 (100%); 538.80 (60%). Anal. calcd. for C_18_H_13_O_9_Re·H_2_O: C 37.43, H 2.62. Found: C 37.98, H 2.15.

### X-ray crystallography of Re–chrysin

Crystals of *fac*-[Re(CO)_3_(chrysin)(MeOH)]0.2MeOH were taken from the mother liquor and mounted at − 143 °C on a Bruker Kappa APEX2 (Bruker Corp., Billerica, Ma, USA) diffractometer equipped with a triumph monochromator using Mo Kα radiation. Unit cell dimensions were determined and refined by using the angular settings of 487 high intensity reflections (> 10 σ(I)) in the range 15 < 2*θ* < 40°. Intensity data were recorded using *φ* and *ω* scans. Crystal presented no decay during the data collection. The frames collected were integrated with the Bruker SAINT software package [46], using a narrow-frame algorithm. Data were corrected for absorption according to the literature [47]. The structure was solved using the Shelxs86 package [48]. Data refinement (full–matrix least–squares methods on *F*^2^) and all subsequent calculations were carried out using Crystals program package version 14.61 build 62.36 [49, 50]. Molecular illustrations with 50% ellipsoid probability were drawn using CAMERON incorporated into crystals [51, 52].

All non-hydrogen atoms were refined anisotropically. Hydrogen atoms were located by difference maps at their expected positions and refined using soft constraints. By the end of the refinement, they were positioned geometrically using riding constraints to bonded atoms. Crystal data as well as details of data collection and structure refinement for the compounds are given in Table 1. Further details on the crystallographic studies as well as atomic displacement parameters are given as Supporting Information in the form of cif files.

**Table 1:** Experimental details

Crystal data	
Chemical formula	C_21_H_21_O_10_Re
*M*_r_	619.59
Crystal system, space group	Triclinic, *P*ī
Temperature (K)	130
*a*, *b*, *c* (Å)	8.2468 (5), 11.0236 (5), 12.8656 (5)
α, β, γ (°)	99.501 (2), 94.8204 (15), 110.312 (2)
*V* (Å^3^)	1069.13 (10)
*Z*	2
Radiation type	Mo *K*α
µ (mm^−1^)	5.74
Crystal size (mm)	0.19 × 0.15 × 0.12
Data collection	
Diffractometer	Bruker Kappa Apex2
Absorption correction	Numerical analytical absorption [47]
*T*_min_, *T*_max_	0.42, 0.50
No. of measured, independent and observed [*I* > 2.0σ(*I*)] reflections	19,819, 4088, 3503
*R*_int_	0.069
(sin θ/λ)_max_ (Å^−1^)	0.615
Refinement	
*R*[*F*^2^ > 2σ(*F*^2^)], *wR*(*F*^2^), *S*	0.041, 0.092, 1.00
No. of reflections	3503
No. of parameters	305
No. of restraints	14
H-atom treatment	H atoms treated by a mixture of independent and constrained refinement
Δ*ρ*_max_, Δ*ρ*_min_ (e Å^−3^)	1.73−1.51

CCDC 2126509 contains the supplementary crystallographic data for this paper. These data can be obtained free of charge via www.ccdc.cam.ac.uk/data_request/cif, or by emailing to data_request@ccdc.cam.ac.uk, or by contacting The Cambridge Crystallographic Data Centre, 12 Union Road, Cambridge CB2 1EZ, UK; Fax: + 44 1223336033.

### In vitro assays

The in vitro assays were performed at a concentration of 100 µM (a stock solution 10 mM in DMSO was used, from which several dilutions were made for the determination of IC_50_ values), at least in triplicate and the standard deviation of absorbance was less than 10% of the mean. The compounds were diluted as 0.1% DMSO under sonication in the appropriate buffer in several dilutions (Table 2).

i. Determination of the reducing activity of the stable radical DPPH [53]

**Table 2:** In vitro % lipoxygenase (LOX %) inhibitory activity of the flavonoids and their Re-complexes. Percent interaction with the stable radical 1,1-diphenylpicrylhydrazyl (DPPH) (RA %)

Compounds	RA % 100 µM at 20 min	RA % 100 µM at 60 min	LOX % 100 µM or IC_50_	AAPH (%) 100 µM
resokaempferol	78	77	90	No
Re–resokaempferol	87	88	37	No
quercetin	84	100	4.75 μΜ	95
Re–quercetin	54	100	83 (35 μΜ)	91
chrysin	3	No	5	No
Re–chrysin	14	19	14	No
naringenin	24	33	5.7 μΜ	No
Re–naringenin	49	54	21	No
[Re(CO)_3_(H_2_O)_3_]^+^	No	No	No	No
Trolox				92
NDGA	91	97	93 (0.45 μΜ)	

Values are means ± SD of three or four different determinations; No: no action under the used experimental conditions

In vitro % inhibition of linoleic acid peroxidation (AAPH %) assay

To an ethanolic solution of DPPH (50 μΜ) in absolute ethanol, an equal volume of the compounds dissolved in DMSO was added (100 μΜ). The mixture was shaken vigorously and allowed to stand for 20 min or 60 min; absorbance at 517 nm was determined spectrophotometrically and the percentage of activity was calculated. All tests were performed in triplicates and the results were averaged (Table 2). NDGA was used as a standard reference compound.

ii. Inhibition of linoleic acid lipid peroxidation [53]

Production of conjugated diene hydroperoxide by oxidation of linoleic acid in an aqueous dispersion is monitored at 234 nm. AAPH is used as a free radical initiator. This assay can be used to follow oxidative changes and to understand the contribution of each tested compound. The experimental procedure follows our previously reported protocol.

In 16 mM linoleic acid sodium salt solution, 10 µL of the tested compounds (final concentration 100 μΜ) was added to the UV cuvette containing 0.05 M phosphate buffer, pH 7.4 preheated to 37 °C. The oxidation reaction was initiated at 37 °C under air by the addition of 40 mM AAPH solution. Oxidation was carried out in the presence of the tested compounds in the assay without antioxidant, lipid oxidation was measured in the presence of the same level of DMSO. The rate of oxidation at 37 °C was monitored by recording the increase in absorption at 234 nm caused by conjugated diene hydroperoxides. Trolox was used as a standard reference compound.

iii. Soybean LOX inhibition study in vitro [53]

The tested compounds dissolved in DMSO (a stock solution 10 mM in DMSO was used) were incubated at RT with sodium linoleate and 0.2 ml of enzyme solution (1/9 × 10^–4^ w/v in saline) in Tris buffer, pH 9, and 10 µL of the tested compounds (final concentration 100 μΜ) were added. The conversion of sodium linoleate to 13-hydroperoxylinoleic acid at 234 nm was recorded and compared with the appropriate standard inhibitor NDGA. The IC_50_ values were calculated for Re–naringenin and Re–quercetin by performing the measurements at several dilutions.

### RT-QuIC assays

Real-time quaking-induced conversion (RT-QuIC) reactions are cell-free assays, currently used to estimate whether compounds can impede the conversion of the normal isoform of the prion protein (PrP^C^) into PrP^Sc^, the abnormally folded, disease-associated, prone to aggregate isoform [54]. Assays were performed as previously described [55]. In brief, 15 μl of cerebrospinal fluid (CSF) from patients with sporadic Creutzfeldt–Jakob disease (sCJD) diluted 10^3^-fold in 1 × phosphate buffered saline (PBS) were mixed with 85 μl of reaction buffer consisting of 5 × PBS (pH 6.9), 170 mM sodium chloride, 1 mM EDTA, 10 μM Thioflavin-T and 0.1 mg/mL recombinant PrP (hamster-sheep chimera sequence). The lead and the labelled compounds were diluted in DMSO and added to a final concentration of 1 and 20 μΜ at the beginning of the reactions, to evaluate whether they would efficiently inhibit the conversion of PrP^C^ to PrP^Sc^ and the aggregation of the later. To rule out any non-specific effects Control reactions, in which CSF from healthy individuals instead of CJD patients was added along with the compounds, and DMSO only reactions, wherein no compound was added were also included. The reactions were setup in 96-well black optical bottom plates and carried out in a FLUO Star OPTIMA (BMG Labtech, Ortenberg. Baden-Württemberg, Germany plate reader at 42 °C for 80 h with intermittent quaking cycles (double-orbital quaking at 600 rpm: one minute, incubation break: one minute). Kinetics of the beta-sheet formation were monitored by Thioflavin T fluorescence (excitation 450 nm, emission 480 nm) every 30 min. To rule out any non-specific effects Control reactions, in which CSF from healthy individuals instead of CJD patients was added, and DMSO only reactions, wherein no compound was added were also included.

## Results and discussion

### Synthesis and characterization

**Scheme 1:**
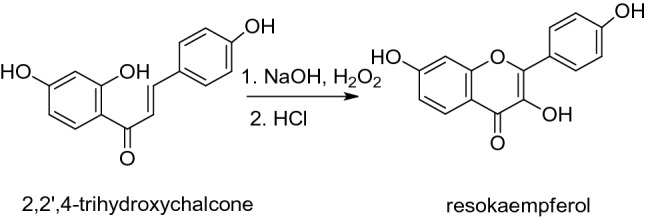
Synthesis of resokaempferol

The preparation of resokaempferol was performed by alkaline cyclization in NaOH solution and subsequent addition of hydrogen peroxide (Scheme 1).

The preparation of the rhenium complexes was performed by reaction of the precursor *fac*-[Re(CO)_3_(sol)_3_]^+^ (sol is water or methanol) with equimolar amounts of the flavones. The complexes were purified by semi-preparative RP-HPLC (Supplementary material).

Characterization by IR spectroscopy showed that the complexes exhibited strong IR *ν*(CO) stretching bands at 2019, 1870 cm^−1^ for Re–resokaempferol*,* 2024, 1949, 1924 cm^−1^ for Re–quercetin*,* 2026, 1888 cm^−1^ for Re–naringenin*,* 2021, 1876 cm^−1^ for Re–chrysin. The ESI-MS spectra of all the rhenium complexes exhibited one major molecular ion at [M-Solvent-H]^−^ m/z.

**Fig. 2:**
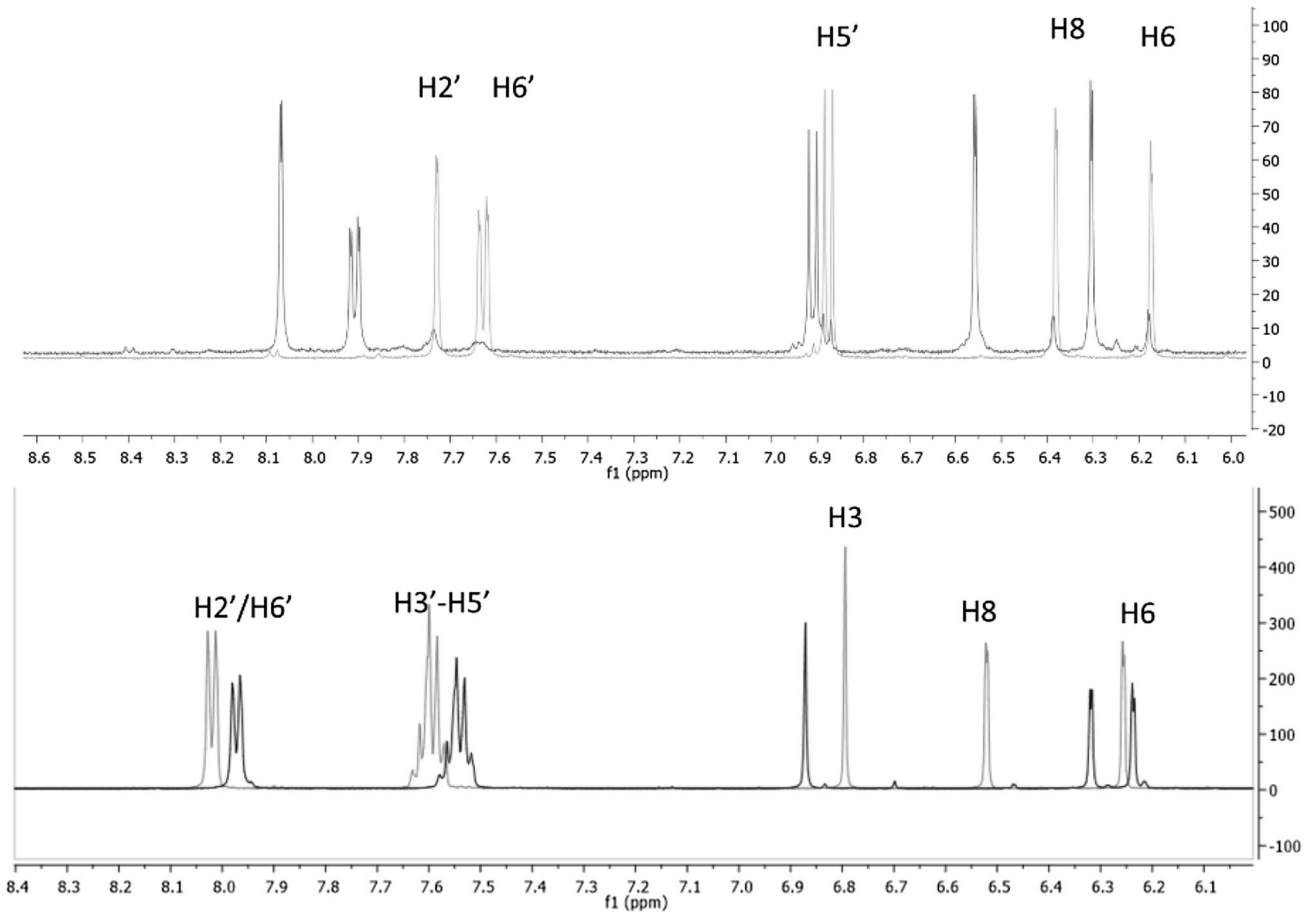
^1^H-NMR comparison of top: quercetin (gray)/Re–quercetin (black); bottom: chrysin (gray)/Re–chrysin (black)

In the ^1^H-NMR spectra of the rhenium complexes of 3-resokaempferol and quercetin, a significant downfield shift of the B flavone ring protons 2’ and 6’ was observed due to the metal coordination at the C-4 carbonyl oxygen and the C-3 phenolic oxygen of the C flavone ring (Fig. 2). In particular, H-2’/H-6’ protons shifted from 8.11 to 8.41 ppm in Re–resokaempferol and from 7.71/7.57 to 8.07/7.92 ppm in Re–quercetin, respectively. Furthermore, in the ^13^C-NMR there was a downfield shift of C-3 from 137 to 150 ppm and of C-4 from 173 to 182 ppm in Re–resokaempferol. Respectively, in Re–quercetin, a shift of C-3 from 137 to 151 ppm and a shift of C-4 from 176 to 182 ppm was observed. The shifts of the Re-coordinated carbonyl ligands are at 198 ppm in both Re–resokaempferol and Re–quercetin. On the other hand, in the rhenium complexes of naringenin and chrysin the shifts of ring B protons remain undisturbed. In the ^1^H-NMR of the Re–chrysin complex (Fig. 2), H-8 proton shifted from 6.52 to 6.32 ppm and in the ^13^C-NMR spectrum C-4 shifted from 182 to 178 ppm, C-5 from 162 to 166 ppm and C-10 from 105 to 108 ppm. The shifts of the Re–coordinated carbonyl ligands are at 197.0, 196.9, 196.7 ppm. In the ^1^H NMR spectrum of the complex Re–naringenin, two isomers were detected, due to the chiral C-2 carbon. In particular, the H-2 proton of the ligand at 5.3 ppm exhibits two signals at 5.2 and 5.3 ppm in the Re-complex and the H-3 protons of the ligand at 3.1 and 2.7 ppm exhibit signals at 2.8/3.2 ppm and 2.8/3.0 ppm in the Re-complex, respectively. In the ^13^C-NMR spectrum of Re–naringenin, C-4 shifted from 196 to 190 ppm, C-5 from 163 to 168 ppm and C-10 from 102 to 107 ppm. The shifts of the Re-coordinated carbonyl ligands are at 197.4, 197, 196.7 ppm.

The stability of the rhenium complexes in degassed DMSO solution was evaluated by HPLC analysis for 1 week, where Re–chrysin and Re–naringenin were found to be > 95% stable. Re–quercetin and Re–resokaempferol were > 95% stable for 48 h. The biological studies were performed from freshly dissolved compounds.

The emission spectra of the complexes in concentration 10 μΜ were recorded using their absorbance maximum for excitation λ. Data are shown in Fig. S1 for Re–resokaempferol and Re–quercetin that exhibited the highest emission. Re–resokaempferol exhibited maxima at 416 and 438 nm after excitation at 346 nm. Re–quercetin exhibited maximum at 525 nm after excitation at 470 nm.

### X-ray crystallography of rhenium–chrysin complex

**Fig. 3:**
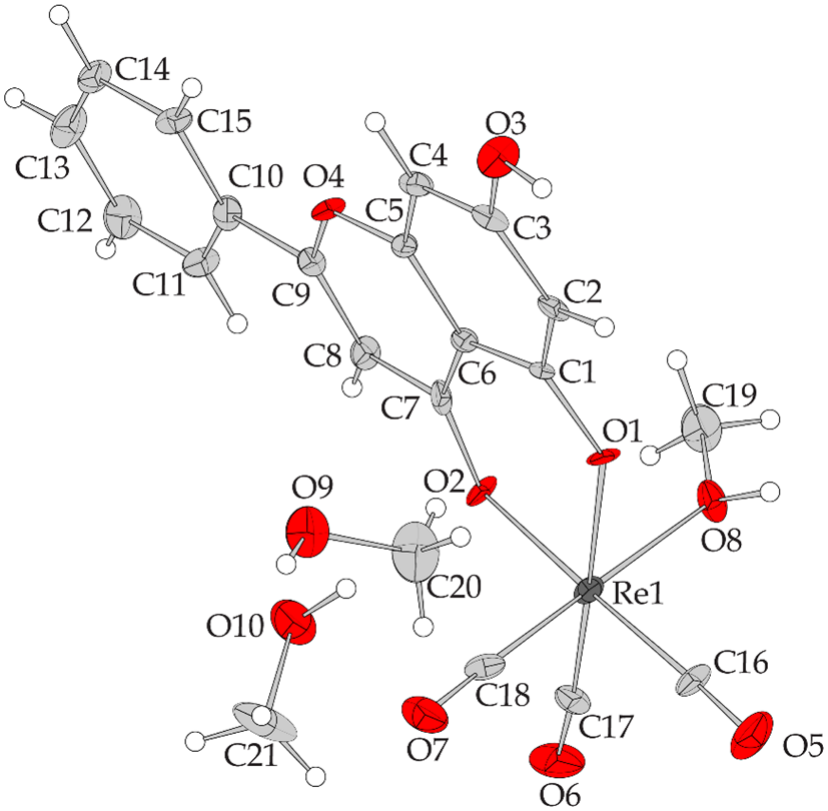
Structure of [Re(CO)_3_(chrysin)(MeOH)], 2(MeOH). Ellipsoids were drawn with 50% probability. Selected bond lengths (Å): Re1—O1 = 2.124 (5); Re1—O2 = 2.092 (5); Re1—O8 = 2.185 (6); Re1—C16 = 1.925 (8); Re1—C17 = 1.921 (9); Re1—C18 = 1.913 (9). Selected angles (.^o^): O1—Re1—O2 = 84.2 (2); O1—Re1—O8 = 81.4 (2); O2—Re1—O8 = 84.4 (2); O2—Re1—C16 = 176.5 (3); O1—Re1—C17 = 174.0 (3); O8—Re1—C18 = 177.3 (3)

A diagram of the structure of complex Re–chrysin is depicted in Fig. 3, bond distances and angles (Table S1) and hydrogen bonding data (Table S2) are included in the Supplementary Material. The complex is mononuclear where chrysin behaves as a bidentate ligand coordinated to rhenium via the carbonyl oxygen atom O(2), and the phenolic oxygen atom O(1) forming a six–membered chelate ring. Additionally, 3 carbon monoxide molecules are coordinated via the carbon atoms C(16, C(17), C(18) in facial orientation and one methanol molecule is coordinated via the oxygen atom O(8). The bond distances and angles are comparable to other structures of rhenium(I)–tricarbonyl coordinated to bidentate (O,O) donor atoms that form a six-membered ring, such as acetylacetonato ligand [56, 57] and oxolinic acid [58], as well as five-membered ring forming bidentate chelators, such as tropolonato [59] and 3-hydroxyflavone [60]. Two solvent molecules of methanol are also present in the structure of the asymmetric unit. Hydrogen bonding interactions between the carbonyl and phenolic oxygen atoms of the ligand, and the oxygen atoms of the alcoholic group of the solvate and ligated methanols are also present.

### In vitro assays

A prominent property of flavonoids is that, through their antioxidant ability, they might provide protection against oxidative stress. The antioxidant activity of flavonoids and other polyphenols is attributed primarily to their ability to scavenge reactive oxygen and nitrogen species [61]. In this study, the rhenium complexes of three flavones and one flavanol were tested as antioxidant agents and inhibitors of lipoxygenase. Antioxidants acting as lipid peroxidation inhibitors could offer health maintenance and compensation of risk factors [62].

The evaluation of the Re-complexes against soybean lipoxygenase LOX was accomplished by the UV-based enzyme assay of Hadjipavlou-Litina et al. [63]. This assay may be used for qualitative or semi-quantitative screen for such activity [64]. Chrysin and Re–chrysin exhibited very low LOX inhibition, which may be explained by the absence of a phenolic moiety in the B-ring of chrysin (Table 2). Significant decrease in LOX inhibition was observed for the other rhenium complexes compared to the respective flavonoids (Table 2). We decided to determine the IC_50_ values for quercetin and Re complex since both present high interaction activity with DPPH after 60 min and high inhibition of lipid peroxidation. Quercetin exhibits high inhibition of LOX with IC_50_ value of 4.75 μM and Re–quercetin IC_50_ of 35 μΜ, under the experimental conditions, respectively. The order of LOX inhibition for the Re-complexes was Re–quercetin > Re–resokaempferol > Re–naringenin. The biological results of Re–quercetin are mainly correlated with the structural characteristics and the presence of quercetin [9, 65].

The antioxidant activity of a compound must be evaluated in a variety of milieus, since factors like solubility or steric hindrance must be considered. Thus, several different assays must be used to avoid conflicts. Also, in each protocol the generation of a different radical is taking place. Two types of experimental approaches have been considered: (i) the assays in which we have the scavenging by hydrogen- or electron donation of a preformed free radical as a marker of antioxidant activity as well as (ii) assays involving the presence of an antioxidant system during the generation of the radical.

The Re-complexes were studied for their antioxidant activity by the use of the 2,2-diphenyl-1-picrylhydrazyl radical (DPPH) at concentration 100 µM after incubation for 20 and 60 min (Table 2). In the DPPH assay, the dominant chemical reaction involved is the reduction of the DPPH radical by single electron transfer (SET) from the antioxidant. Phenolic compounds, e.g. NDGA giving phenoxide anions are effective antioxidants. All the Re-complexes (with the exception of Re–chrysin) interact with the free stable radical DPPH. Re–quercetin presents lower antioxidant activity than quercetin at 20 min. On the contrary, the reducing abilities of Re–naringenin and Re–resokaempferol are higher compared to the flavonoids. The antioxidant activities of resokaempferol, naringenin as well as of Re–resokaempferol and Re–naringenin complexes do not seem to be influenced over the time. The measured antioxidant properties for chrysin and its complex are insignificant, likely due to the absence of a hydroxyl moiety in the B-ring. In the literature, Zn complexes of chrysin and quercetin exhibited enhanced radical scavenging potential [26].

The Re–flavonoid complexes were assessed in vitro for their ability to inhibit peroxidation of linoleic acid in the presence of AAPH, an azo compound generating free radicals through spontaneous thermal decomposition. AAPH has been used in in vitro model of oxidative stress. The activity of the peroxyl radicals produced by the action of AAPH greatly resembles cellular activities such as lipid peroxidation and used to mimic the oxidative stress state.

Re–quercetin is the only complex with anti-lipid peroxidation activity, which is equipotent to that of quercetin. Quercetin affects immunity and inflammation by acting mainly on leukocytes and targeting many intracellular signaling kinases and phosphatases, enzymes and membrane proteins often crucial for a cellular specific function and it was found to inhibit in particular rabbit reticulocyte 15-lipoxygenase-1 (EC 1.13.11.33) [66] and recombinant human 5-lipoxygenase (EC 1.13.11.34) [67].

Most of the LOX inhibitors present antioxidant activity or act as free radical scavengers [68], since lipoxygenation occurs via a carbon centered radical. Herein the anti-lipid peroxidation results for the Re–quercetin complex support the anti-lipoxygenase activity. Analogous results of free quercetin and other flavonoids have been reported in the literature [9, 65].

Free radicals are highly implicated in lipoxygenase inhibition, inflammation, cancer and neurodegeneration. Thus, compounds possessing these activities in combination with antioxidant activity might offer to the prophylaxis or therapy of these diseases.

### RT-QuIC assays

RT-QuIC assays provide a fast and efficient way to screen compounds for their anti-aggregation properties in a cell-free setting and the use of RT-QuIC and similar approaches has expanded considerably throughout the past few years both for diagnosis and for drug development [69]. The free flavones and their rhenium complexes were used in RT-QuIC assays to evaluate their anti-aggregation properties in sCJD. sCJD is the most common form of Transmissible Spongiform Encephalopathy and is associated with the conversion of the cellular form of the prion protein (PrP^C^) to its disease associated and aggregation-prone counterpart (PrP^Sc^).

In our setting, PrP^Sc^ from the CSF of patients with confirmed sCJD was used as the seed for the conversion of recombinant PrP (rPrP), which is soluble and cannot form aggregates. Throughout repeating RT-QuIC cycles, the tertiary structure of rPrP molecules is altered and novel aggregation-prone, PrP^Sc^-like molecules are formed. This structural conversion is monitored via the incorporation of Thioflavin T and the recording of the fluorescence emission [70].

**Fig. 4:**
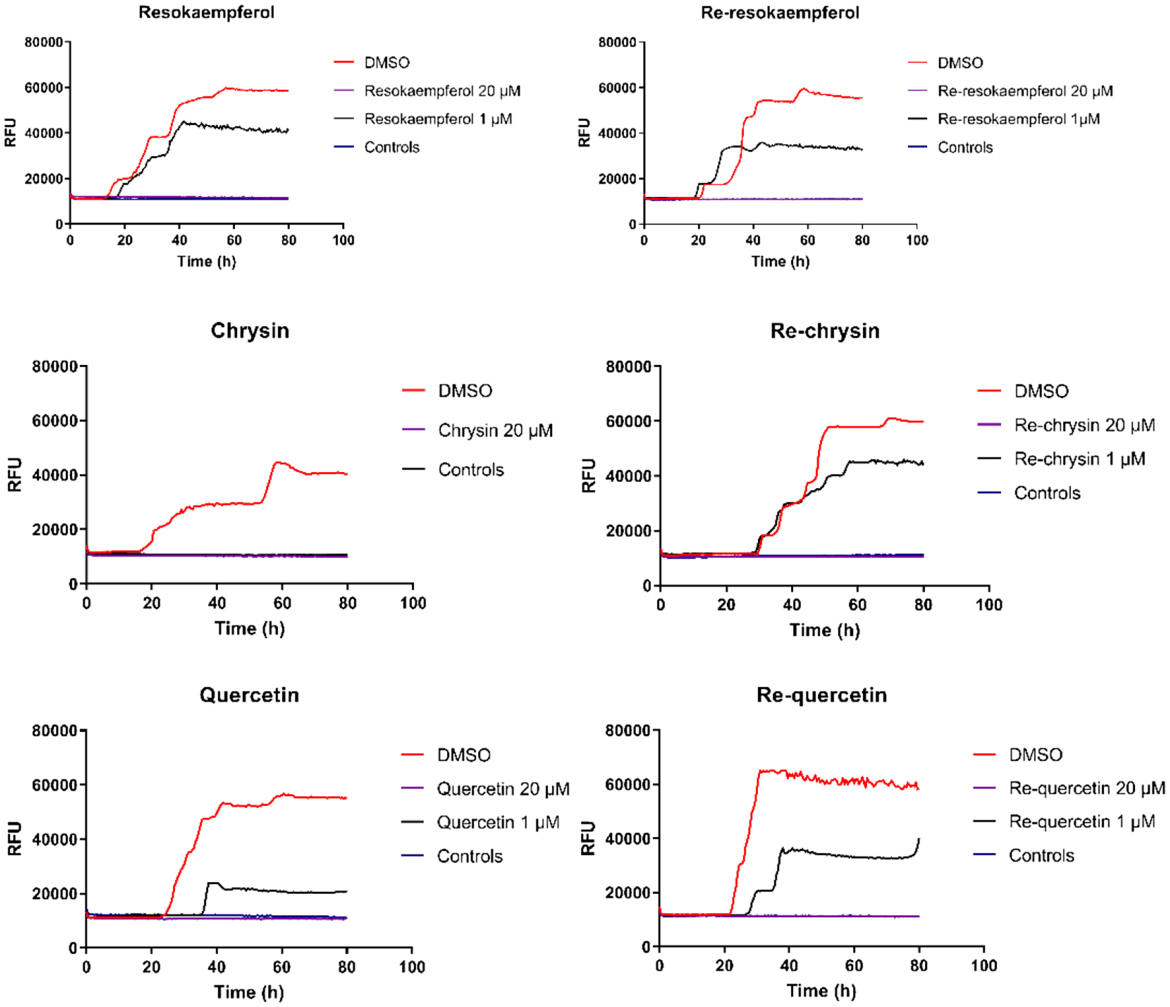
RT-QuIC assays were performed for each compound for 80 h. Thioflavin T emission was recorded every 30 min and expressed as relative fluorescence units (RFU). Each reaction consisted of the compound (diluted in DMSO), cerebrospinal fluid (CSF) from healthy controls or patients with sporadic Creutzfeldt–Jakob disease (sCJD) diluted in Phosphate Buffered Saline (PBS) containing 170 mM sodium chloride, 1 mM EDTA, 10 μM Thioflavin-T and 0.1 mg/mL recombinant PrP. For all the compounds except Chrysine two different final concentrations (1 and 20 μM) were tested. The higher concentration of the compounds (20 μΜ) was also added to control reactions, in which CSF from healthy individuals that did not contain the pathogenic PrP^Sc^ isoform was used as the seed. Furthermore, DMSO only control reactions (DMSO), in which DMSO with a final concentration corresponding to the one obtained with the higher compound concentration and CSF from sCJD patients were used as positive control reactions

The compounds were initially diluted in DMSO and were added to the reaction mix in a final concentration of 20 μM or 1 μΜ (with the exception of Chrysin that was only tested at a final concentration of 20 μM). Each assay was repeated in triplicates and the fluorescence data (relative fluorescence data, RFU) were recorded every 30 min for 80 h (Fig. 4). As expected, in control assays in which only DMSO was added, PrP^C^ was successfully converted to PrP^Sc^, yielding the highest RFU readouts. On the other hand, all the compounds successfully inhibited the conversion of PrP^C^ to the disease-associated isoform PrP^Sc^ when added at a final concentration of 20 μM. In this case, the RFU readouts upon addition of the compounds remained low throughout the assay and matched those of the Control reactions wherein the pathogenic seed had not been added. This effect appears to be dose related: even though rigorous dose–response experiments were not performed, when lower concentrations of the compounds were added to the reaction mix, PrP^C^ conversion was only moderately inhibited. Dose–response experiments would also provide more detailed information on the effect chemical modification has on the anti-aggregation properties of the compounds. Our initial data indicate that the conversion of the free flavonoids to their rhenium complexes did not result in loss of their ability to inhibit the formation of PrP^Sc^, indicating that these complexes retain their activity. Preliminary data also indicates that the compounds can solubilize already formed aggregates. In modified RT-QuIC assays, we added the compounds after the PrPSc aggregates had been formed, as evidenced by the fluorescence readouts. In this setting, we observed that the compounds produced a gradual decrease of the fluorescence, indicating a reduction of the PrP^Sc^ aggregates (Fig. S2). These data imply that the compounds may not only have a protective role, but also a therapeutic.

Even though the conversion mechanism has not yet been defined, it has been suggested that similar conversion and aggregation mechanisms could be involved in other neurodegenerative proteinopathies as well, including Alzheimer’s and Parkinson’s [71] diseases thus indicating that the compounds developed could be useful as theranostics in the whole spectrum of neurodegenerative diseases (Fig. 4).

## Conclusions

The synthesis of the rhenium–tricarbonyl complexes of a variety of flavonoids that belong to the class of either 3-flavonols, such as resokaempferol and quercetin, of flavones, such as chrysin and of flavanones, such as naringenin was reported. Based on spectroscopic data the complexation takes place via the carbonyl and the neighboring hydroxyl group, either (O4-O3) or (O4-O5) and the complexes exhibit comparable stability in solution. In Re–quercetin, NMR data indicate that the coordination via the carbonyl O4 and 3-hydroxyl O occurs. All complexes exhibited anti-oxidant properties against the DPPH radical, with the exception of Re–chrysin. LOX inhibition was observed for the Re-complexes of resokaempferol, quercetin, naringenin, which was lower compared to the free flavonoid. Re–quercetin exhibited the highest LOX inhibition and antioxidant and anti-lipid peroxidation properties among the complexes. Furthermore, all the tested compounds blocked de novo abnormal PrP formation and aggregation in cell free systems. Prompted by these promising results, future studies to determine the ability of Re–quercetin and Re–resokaempferol as fluorescent probes to detect amyloid plaques are envisioned. In addition, these complexes could be used for the design of multi-targeted Re–tricarbonyl “2 + 1” complexes for therapy as well as “2 + 1” technetium-99 m-tricarbonyl complexes for amyloid-targeted scintigraphy.

## Supplementary Information

Below is the link to the electronic supplementary material.

Supplementary file1 (PDF 534 KB)

Supplementary file2 (PDF 121 KB)

## Abbreviations

AAPH: 2,2'-Azobis(2-amidinopropane) dihydrochloride
sCJD: Creutzfeldt–Jakob disease
CSF: Cerebrospinal fluid
DMSO: *N*,*N*-Dimethylsulfoxide
DPPH: 1,1-Diphenyl-2-picrylhydrazyl
EDTA: Ethylenediamine tetraacetic acid
ESI-MS: Electron-spray ionization mass spectrometry
Fl: Flavonoid
HPLC: High-pressure liquid chromatography
IC_50_: Half-maximal inhibitory concentration
IR: Infrared spectrometry
LOX: Lipoxygenase
NDGA: Nordihydroguaiaretic acid
PrP^C^: Cellular prion protein
PrP^Sc^: Scrapie isoform of the prion protein
rPrP: Recombinant PrP
RT-QuIC: Real-time quaking-induced conversion
