# Supplementary material for: Synthesis, structural characterization and study of antioxidant and anti-PrPSc properties of flavonoids and their rhenium(I)–tricarbonyl complexes
Source: J Biol Inorg Chem. 2023 Jan 25;28(2):235–47. doi: 10.1007/s00775-022-01986-9 (PMC9981504; doi:10.1007/s00775-022-01986-9)
Supplement: Supplementary file 1 — Supplementary file1 (PDF 534 KB) [file 775_2022_1986_MOESM1_ESM.pdf]

Supplementary material

Synthesis, structural characterization and study of antioxidant and anti-PrP^{Sc} properties of flavonoids and their rhenium(I)-tricarbonyl complexes.

Pigi Glykofridi [1], Vassiliki-Eleni Tziouri[1], Konstantinos Xanthopoulos[1], Maria-Eirini Vlachou[1], Susana Correia[2], Anna-Lisa Fischer[2], Katrin Thüne[2], Antonios Hatzidimitriou[3], Inga Zerr[2], Matthias Schmitz[2], Theodoros Sklaviadis,[1] Dimitra Hadjipavlou-Litina[1], Dionysia Papagiannopoulou[1]

[1]Laboratories of Pharmaceutical Chemistry and of Pharmacology, School of Pharmacy, Faculty of Health Sciences, Aristotle University of Thessaloniki

[2]Department of Neurology, University Medical Center Göttingen and the German Center for Neurodegenerative Diseases (DZNE), Göttingen, Germany

[3]Laboratory of Inorganic Chemistry, Department of Chemistry, Faculty of Sciences, Aristotle University of Thessaloniki

1. HPLC chromatograms of the purified complexes

Re-resokaempferol (System II)

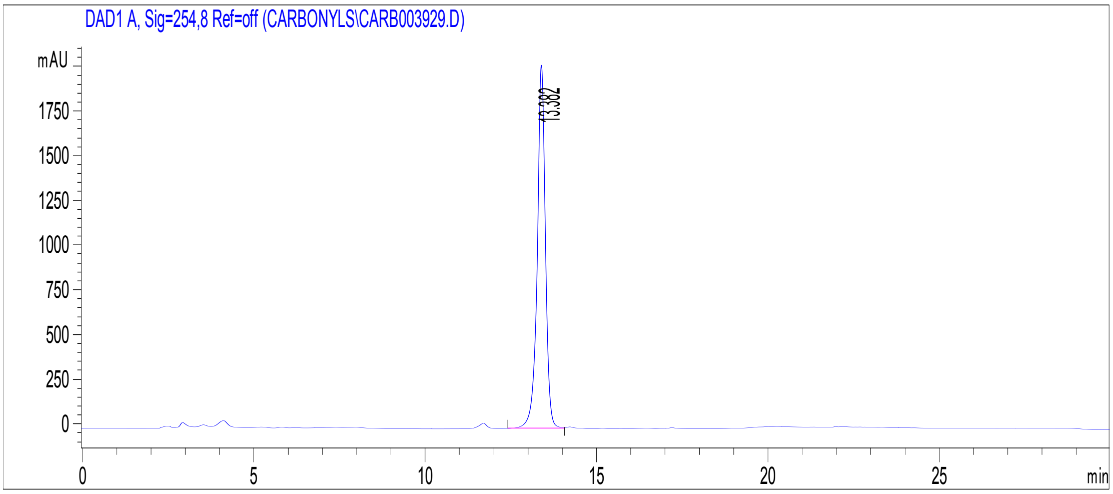

Re-quercetin (System I)

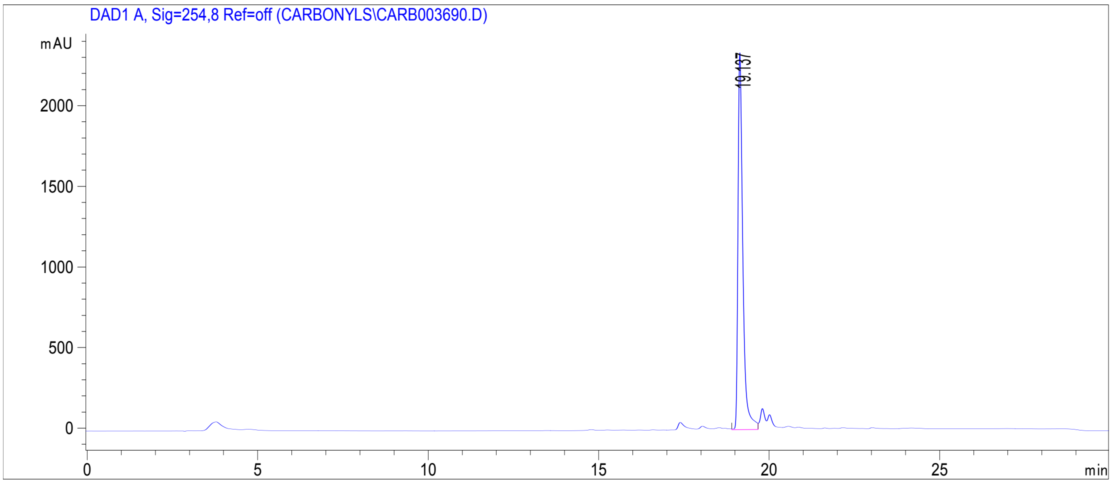

Re-chrysin (System I)

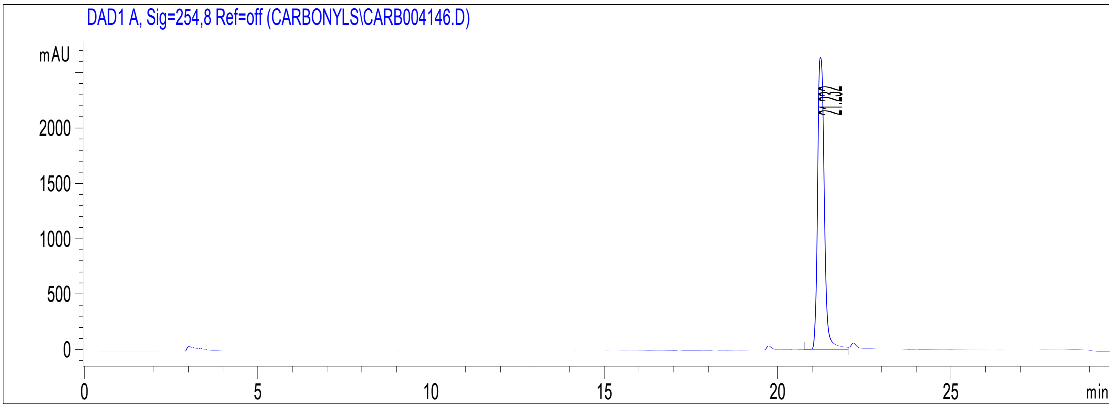

Re-naringenin (System II)

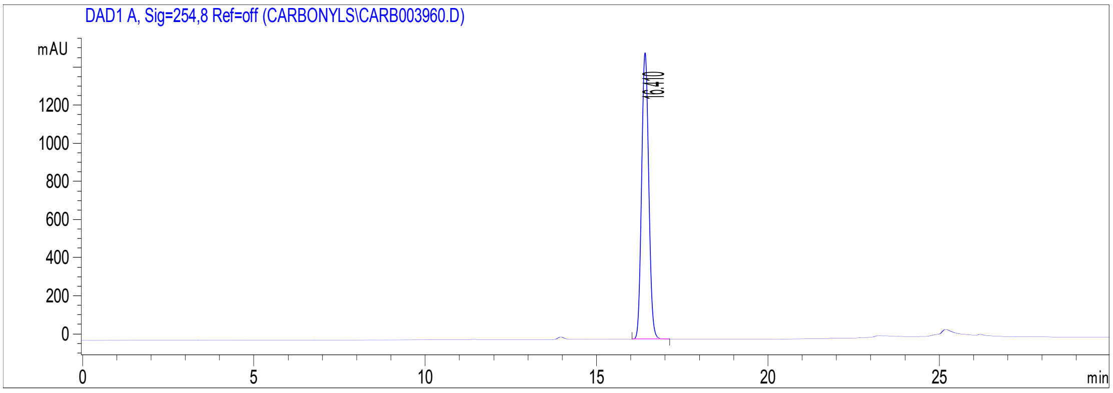

2. NMR Spectra

Re-resokaempferol

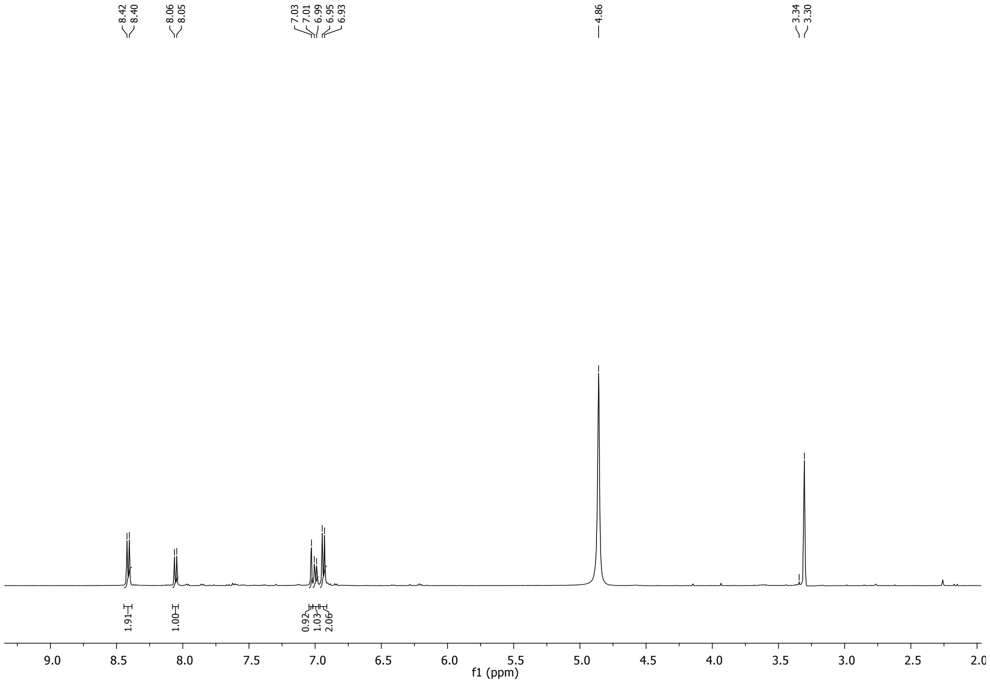

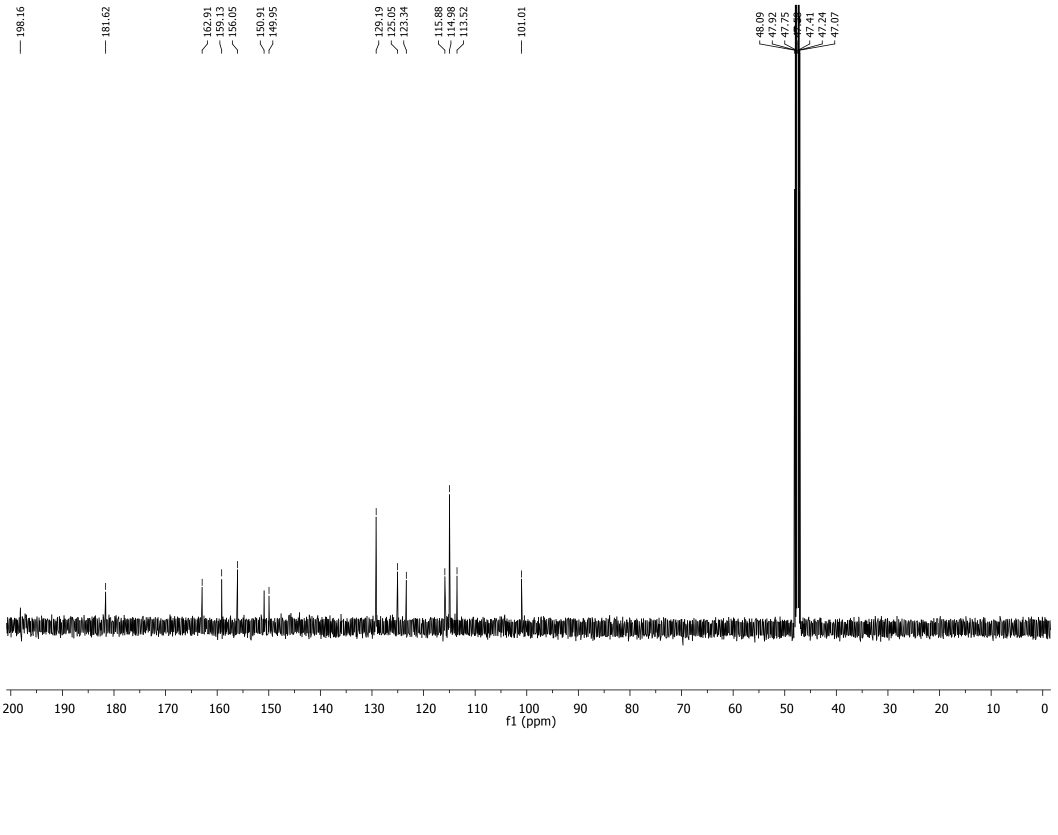

Re-quercetin

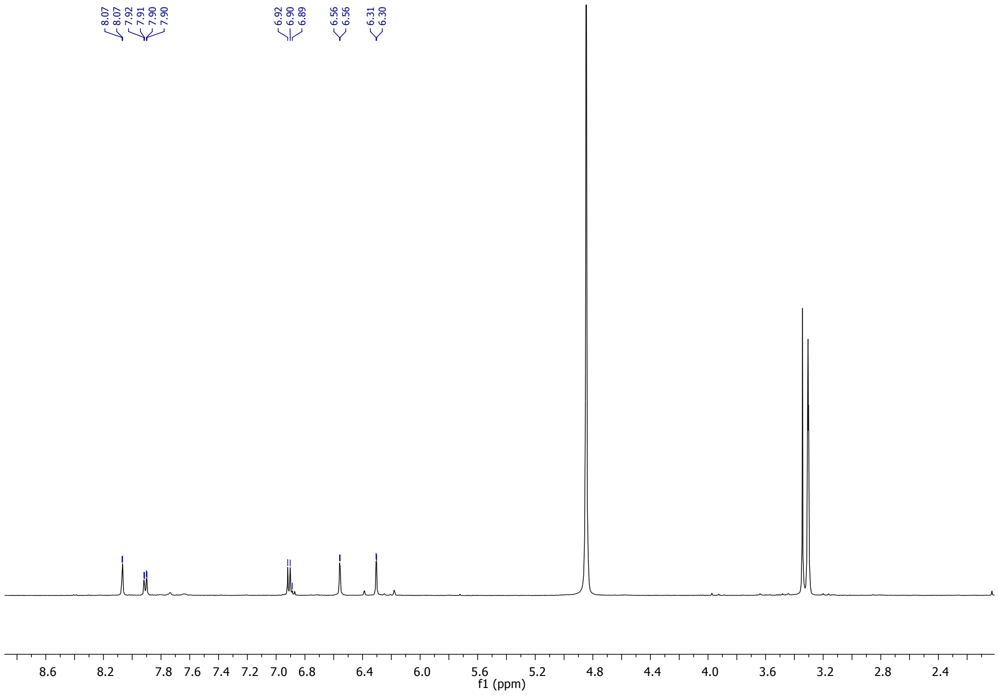

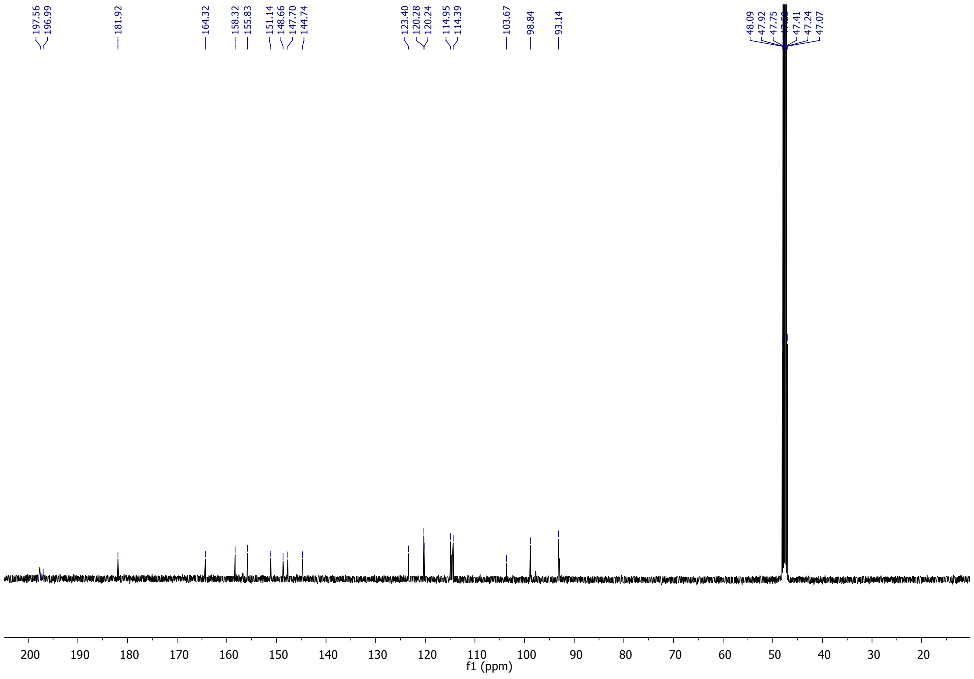

Re-chrysin

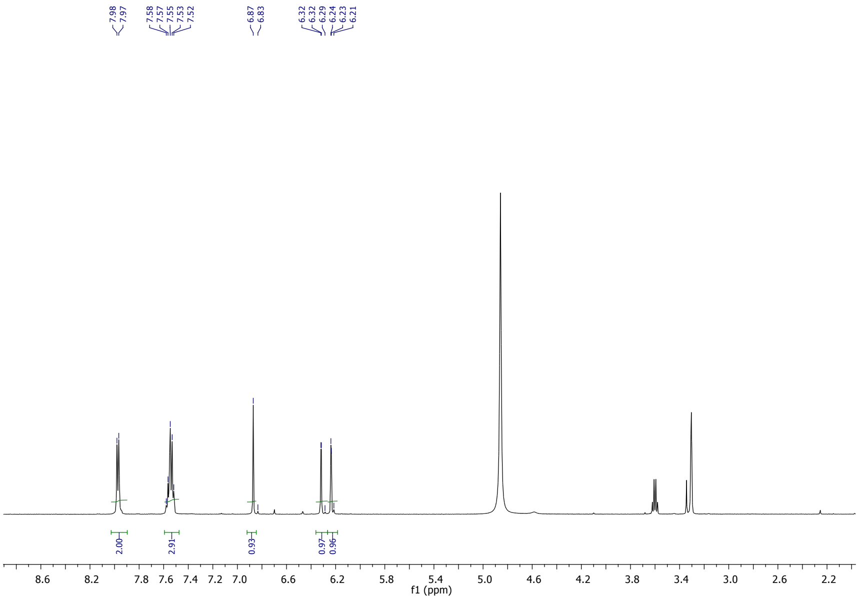

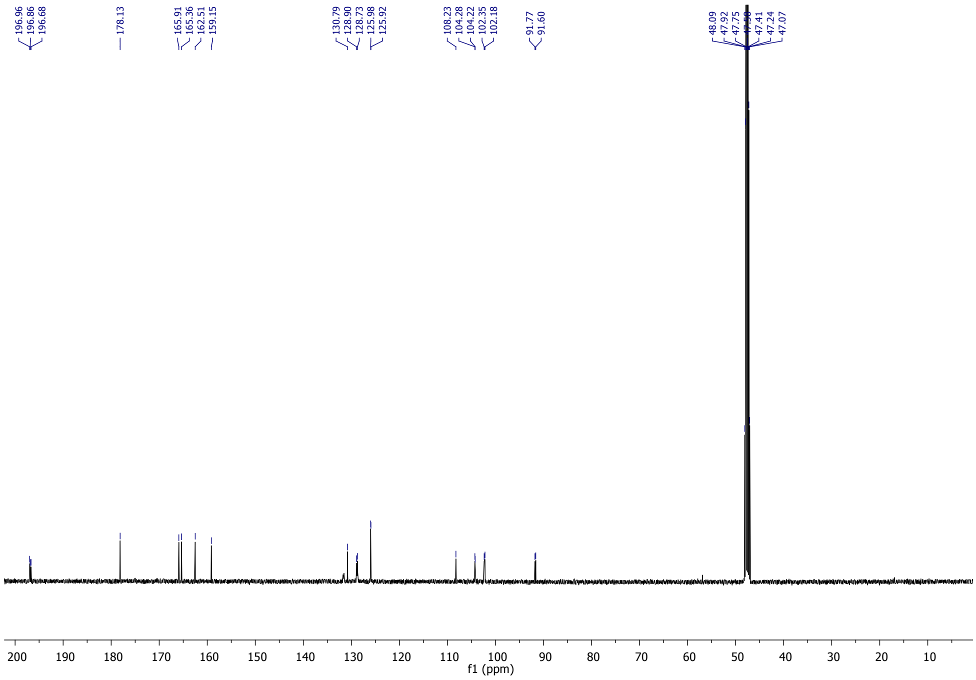

Re-naringenin

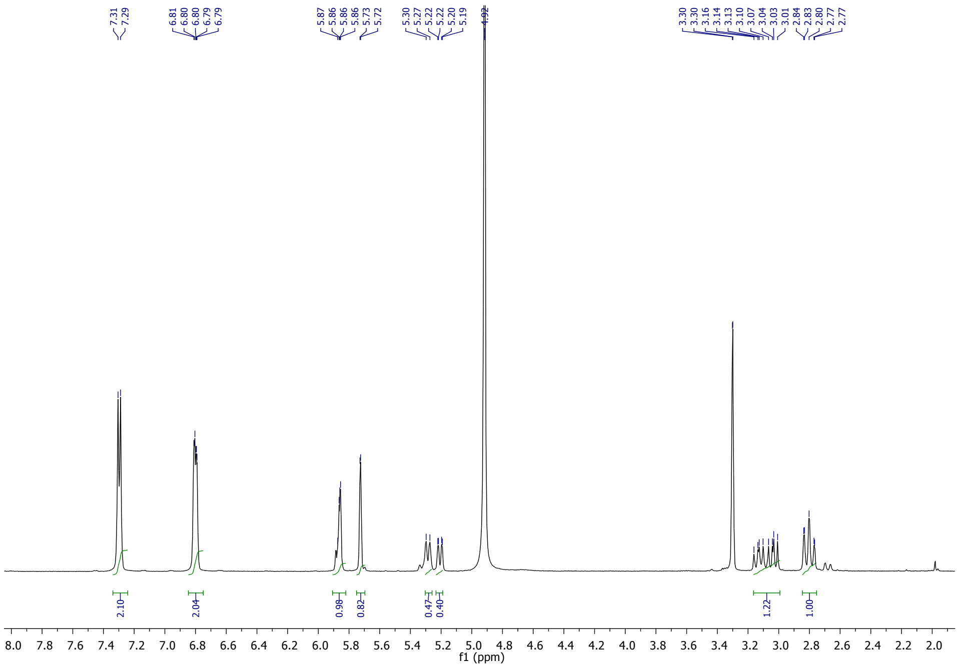

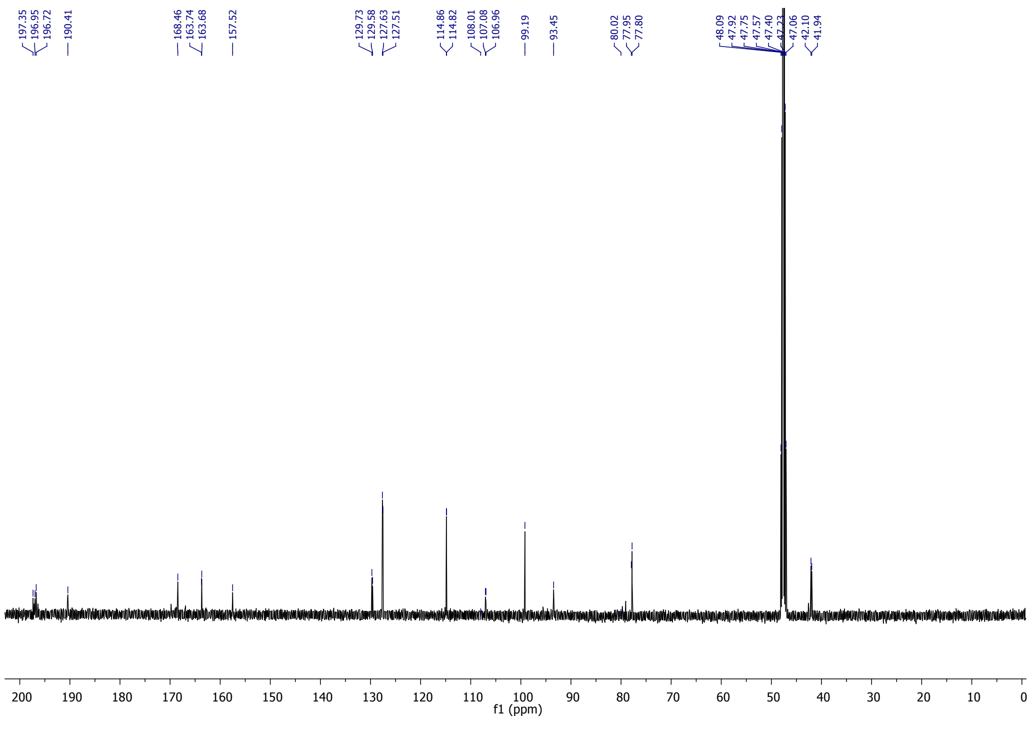

Table S1. Selected geometric parameters (Å, º) of Re-chrysin.

Re1—O1	2.124 (5)	C1—C2	1.372 (11)
Re1—O2	2.092 (5)	C1—C6	1.436 (11)
Re1—O8	2.185 (6)	C2—C3	1.384 (11)
Re1—C16	1.925 (8)	C3—C4	1.386 (12)
Re1—C17	1.921 (9)	C4—C5	1.378 (11)
Re1—C18	1.913 (9)	C5—C6	1.414 (10)
O1—C1	1.328 (9)	C6—C7	1.421 (11)

O2—C7	1.304 (10)	C7—C8	1.435 (10)
O3—C3	1.364 (10)	C8—C9	1.351 (11)
O4—C5	1.372 (9)	C9—C10	1.483 (11)
O4—C9	1.345 (10)	C10—C11	1.405 (12)
O5—C16	1.127 (10)	C10—C15	1.389 (12)
O6—C17	1.143 (11)	C11—C12	1.381 (12)
O7—C18	1.141 (11)	C12—C13	1.372 (13)
O8—C19	1.433 (11)	C13—C14	1.396 (13)
O9—C20	1.411 (13)	C14—C15	1.390 (11)
O10—C21	1.400 (12)		
O1—Re1—O2	84.2 (2)	C3—C4—C5	117.3 (7)
O1—Re1—O8	81.4 (2)	C4—C5—O4	114.8 (7)
O2—Re1—O8	84.4 (2)	C4—C5—C6	122.9 (7)
O1—Re1—C16	96.9 (3)	O4—C5—C6	122.3 (7)
O2—Re1—C16	176.5 (3)	C1—C6—C5	117.6 (7)
O8—Re1—C16	92.6 (3)	C1—C6—C7	125.6 (7)
O1—Re1—C17	174.0 (3)	C5—C6—C7	116.8 (7)
O2—Re1—C17	90.6 (3)	C6—C7—O2	125.4 (7)
O8—Re1—C17	95.0 (3)	C6—C7—C8	118.5 (7)
C16—Re1—C17	88.0 (4)	O2—C7—C8	116.1 (7)
O1—Re1—C18	96.8 (3)	C7—C8—C9	120.4 (8)
O2—Re1—C18	97.5 (3)	C8—C9—O4	122.0 (7)
O8—Re1—C18	177.3 (3)	C8—C9—C10	124.1 (8)
C16—Re1—C18	85.6 (4)	O4—C9—C10	113.9 (7)
C17—Re1—C18	87.0 (4)	C9—C10—C11	119.4 (8)
Re1—O1—C1	131.0 (5)	C9—C10—C15	120.9 (8)
Re1—O2—C7	130.4 (5)	C11—C10—C15	119.7 (8)
C5—O4—C9	119.9 (6)	C10—C11—C12	119.5 (8)
Re1—O8—C19	129.8 (5)	C11—C12—C13	120.8 (9)
O1—C1—C2	119.9 (7)	C12—C13—C14	120.3 (8)
O1—C1—C6	121.3 (7)	C13—C14—C15	119.6 (8)
C2—C1—C6	118.9 (7)	C14—C15—C10	120.1 (8)
C1—C2—C3	121.2 (7)	Re1—C16—O5	177.4 (8)
C2—C3—O3	121.9 (7)	Re1—C17—O6	174.8 (8)
C2—C3—C4	122.0 (8)	Re1—C18—O7	178.0 (8)
O3—C3—C4	116.0 (7)		

Table S2. Hydrogen-bond geometry (Å, º) of *fac*-[Re(CO)$_3$(chrysin)(MeOH)

D—H···*A*	*D*—H	H···*A*	*D*···*A*	*D*—H···*A*
O3—H171···O9^{ii}	0.82	1.84	2.645 (13)	167 (12)
O8—H211···O1^{iii}	0.82	1.77	2.577 (13)	168 (11)
O9—H291···O10	0.82	1.95	2.725 (13)	157 (12)
O10—H321···O2	0.82	2.10	2.913 (13)	170 (13)

Symmetry codes: (ii) *x*+1, *y*+1, *z*; (iii) −*x*+1, −*y*+1, −*z*.

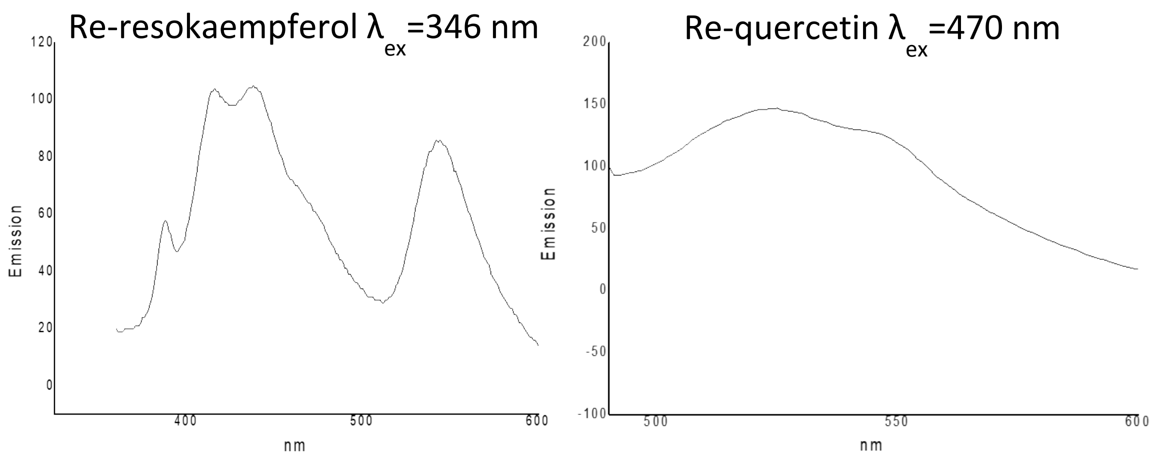

Fig S1. Emission spectra of Re-resokaempferol (excitation at 346 nm) and Re-quercetin (excitation at 470 nm) at 10 μM concentration.

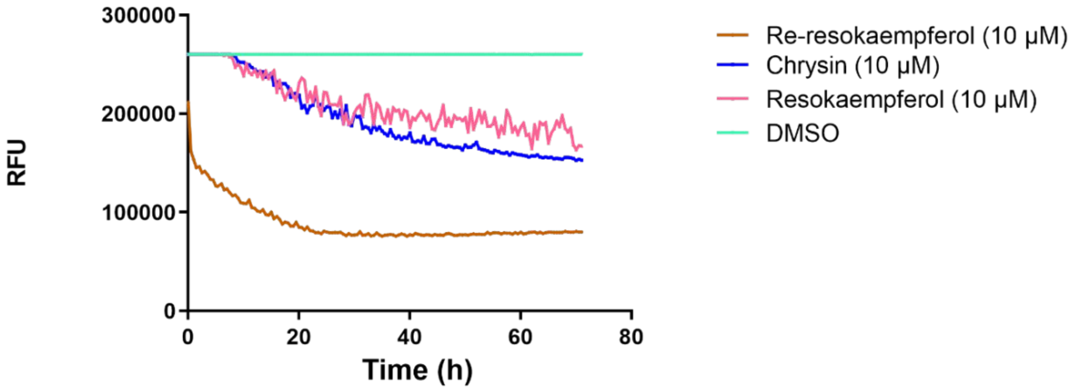

Fig. S2 Modified RT-QuIC assays were performed for Chrysin, Resokaempferol and Re-resokaempferol to assess their ability to disaggregate already formed fibrils. To this end, the DMSO-diluted compounds (or DMSO only in the control DMSO reaction, in a concentration equal to the reactions where the compounds were added) were added to the reaction mix after PrP aggregates had been formed and Thioflavin T emission was recorded every 30 min and expressed as relative fluorescence units (RFU) for the ensuing 80 h. Each reaction consisted of the compound (diluted in DMSO) and cerebrospinal fluid (CSF) from patients with sporadic Creutzfeldt Jakob Disease (sCJD) diluted in Phosphate Buffered Saline (PBS) containing 170 mM sodium chloride, 1 mM EDTA, 10 μM Thioflavin T and 0.1 mg/mL recombinant PrP. The compounds reduce fluorescence in a time-dependent manner, indicating disaggregation of the already formed aggregates.
