# Supplementary material for: Synthesis, structural characterization and study of antioxidant and anti-PrPSc properties of flavonoids and their rhenium(I)–tricarbonyl complexes
Source: J Biol Inorg Chem. 2023 Jan 25;28(2):235–47. doi: 10.1007/s00775-022-01986-9 (PMC9981504; doi:10.1007/s00775-022-01986-9)

checkCIF/PLATON report

Structure factors have been supplied for datablock(s) I

THIS REPORT IS FOR GUIDANCE ONLY. IF USED AS PART OF A REVIEW PROCEDURE FOR PUBLICATION, IT SHOULD NOT REPLACE THE EXPERTISE OF AN EXPERIENCED CRYSTALLOGRAPHIC REFEREE.

No syntax errors found. CIF dictionary Interpreting this report

Datablock: I

```
Bond precision:  C-C = 0.0125 A          Wavelength=0.71073

Cell:          a=8.2468(5)       b=11.0236(5)       c=12.8656(5)
               alpha=99.501(2)   beta=94.8204(15)   gamma=110.312(2)
Temperature:   130 K
```

	Calculated	Reported
Volume	1069.12(9)	1069.13(10)
Space group	P -1	P -1
Hall group	-P 1	?
Moiety formula	C19 H13 O8 Re, 2(C H4 O)	C21 H21 O10 Re1
Sum formula	C21 H21 O10 Re	C21 H21 O10 Re1
Mr	619.59	619.59
Dx,g cm-3	1.925	1.925
Z	2	2
Mu (mm-1)	5.739	5.739
F000	604.0	604.0
F000’	602.35	
h,k,lmax	10,13,15	10,13,15
Nref	4155	4088
Tmin,Tmax	0.370,0.502	0.420,0.500
Tmin’	0.323	

```
Correction method= # Reported T Limits: Tmin=0.420 Tmax=0.500
AbsCorr = NUMERICAL

Data completeness= 0.984          Theta(max)= 25.918

R(reflections)= 0.0408( 3503)     wR2(reflections)= 0.1004( 3503)

S = 1.000                  Npar= 305
```

The following ALERTS were generated. Each ALERT has the format
test-name_ALERT_alert-type_alert-level.
Click on the hyperlinks for more details of the test.

Alert level C

```
PLAT342_ALERT_3_C Low Bond Precision on  C-C Bonds ...............     0.0125 Ang.
PLAT911_ALERT_3_C Missing FCF Refl Between Thmin & STh/L=    0.600         19 Report
```

Alert level G

```
PLAT002_ALERT_2_G Number of Distance or Angle Restraints on AtSite          8 Note
PLAT003_ALERT_2_G Number of Uiso or Uij Restrained non-H Atoms ...          2 Report
PLAT042_ALERT_1_G Calc. and Reported MoietyFormula Strings  Differ     Please Check
PLAT153_ALERT_1_G The s.u.'s on the Cell Axes   are Equal ..(Note)     0.0005 Ang.
PLAT808_ALERT_5_G No Parseable SHELXL Style Weighting Scheme Found     Please Check
PLAT860_ALERT_3_G Number of Least-Squares Restraints .............         14 Note
PLAT882_ALERT_1_G No Datum for _diffrn_reflns_av_unetI/netI ......     Please Do !
PLAT912_ALERT_4_G Missing # of FCF Reflections Above STh/L=  0.600         60 Note
PLAT929_ALERT_5_G No Weight Pars,Obs and Calc R1,wR2,S not Checked          ! Info
PLAT960_ALERT_3_G Number of Intensities with I  <   - 2*sig(I) ...         10 Check
```

```
   0 ALERT level A = Most likely a serious problem - resolve or explain
   0 ALERT level B = A potentially serious problem, consider carefully
   2 ALERT level C = Check. Ensure it is not caused by an omission or oversight
  10 ALERT level G = General information/check it is not something unexpected

   3 ALERT type 1 CIF construction/syntax error, inconsistent or missing data
   2 ALERT type 2 Indicator that the structure model may be wrong or deficient
   4 ALERT type 3 Indicator that the structure quality may be low
   1 ALERT type 4 Improvement, methodology, query or suggestion
   2 ALERT type 5 Informative message, check
```

It is advisable to attempt to resolve as many as possible of the alerts in all categories. Often the minor alerts point to easily fixed oversights, errors and omissions in your CIF or refinement strategy, so attention to these fine details can be worthwhile. In order to resolve some of the more serious problems it may be necessary to carry out additional measurements or structure refinements. However, the purpose of your study may justify the reported deviations and the more serious of these should normally be commented upon in the discussion or experimental section of a paper or in the "special_details" fields of the CIF. checkCIF was carefully designed to identify outliers and unusual parameters, but every test has its limitations and alerts that are not important in a particular case may appear. Conversely, the absence of alerts does not guarantee there are no aspects of the results needing attention. It is up to the individual to critically assess their own results and, if necessary, seek expert advice.

Publication of your CIF in IUCr journals

A basic structural check has been run on your CIF. These basic checks will be run on all CIFs submitted for publication in IUCr journals (*Acta Crystallographica*, *Journal of Applied Crystallography*, *Journal of Synchrotron Radiation*); however, if you intend to submit to *Acta Crystallographica Section C* or *E* or *IUCrData*, you should make sure that full publication checks are run on the final version of your CIF prior to submission.

Publication of your CIF in other journals

Please refer to the *Notes for Authors* of the relevant journal for any special instructions relating to CIF submission.

PLATON version of 30/01/2018; check.def file version of 30/01/2018

Datablock I - ellipsoid plot

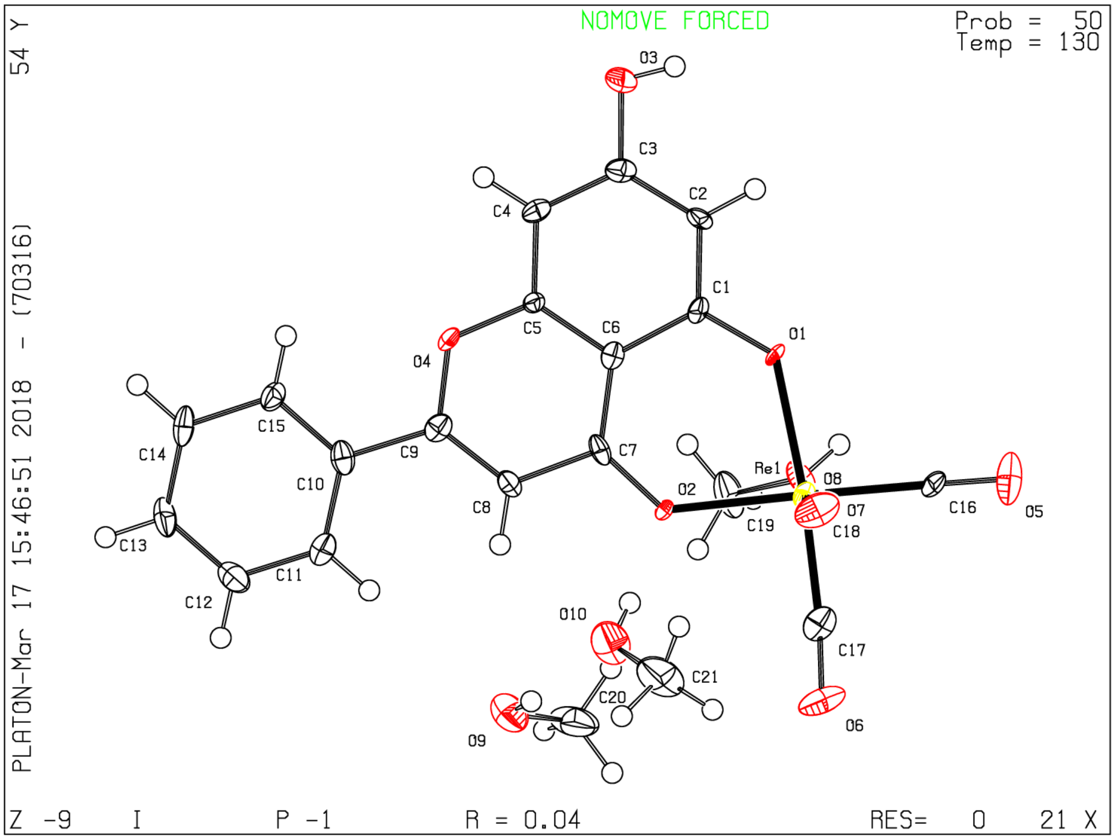

Supplement: Supplementary file 2 — Supplementary file2 (PDF 121 KB) [file 775_2022_1986_MOESM2_ESM.pdf]
